# Traumatic Injuries of the Brain and Its Membranes

**Published:** 1898-02

**Authors:** 


### Books and Magazines.

---

**Traumatic Injuries of the Brain and its Membranes,** With a special study of pistol shot wounds of the head in their medico-legal and surgical relations. By Charles Phelps, M. D., Surgeon to Bellevue and St. Vincent's Hospitals. 8 Vo. Five hundred and eighty-two pages; forty-nine illustrations. Price, cloth, $5.00. D. Appleton & Co., publishers, New York City, 1897.

This work treats only of traumatic injuries of the brain and its membranes, and does not consider diseased conditions of these structures.

Part first treats of cranial fractures, their classification, complications, symptomotology, etc. The author's classification and description of the different forms of fracture are excellent. In speaking of fracture of the vault the author says: "The very frequent coincidence of fracture with intracranial lesion, has led to much confusion in symptomotology and consequent prognosis. Loss of consciousness and variations of pulse, temperature and respiration, with other undoubted indications of intracranial complications, are still enumerated among the symptoms of fracture. These inaccuracies are of consequence, since a lack of well-defined conception of the nature of lesions, or of the significance of symptoms begets errors of treatment. * * * It may be briefly stated that fracture of the vault is to be recognized by tactile or visual sense; that these methods are always practicable; that no others are defensible; and that there is no justification for the neglect to resort to both when one is insufficient for exact diagnosis. * * * This covers the whole ground of diagnosis—tactile or visual examination, and, if necessary for that purpose, unhesitating and sufficient incision down to the cranial surface." As to the prognosis, the author says: "It is therefore true, that in themselves cranial fractures are important only in exceptional cases. Their prognosis is really the prognosis of their complications. Neither the shock of an uncomplicated fracture nor the hemorrhage from the osteal vessels is ever fatal."

The intracranial traumatic lesions, the author says, may be classified primarily as: hemorrhages; thromboses of sinuses; contusions; lacerations; and their sequelæ as: meningeal and parenchymatous inflammations, which are usually, if not invariably, of a septic character; and atrophy. The symptomatology of the various traumatic brain lesions is treated in extenso, the author devoting more than seventy pages to the discussion of this particular subject. The chapters on diagnosis, prognosis, and treatment are also quite full and very satisfactory. A most unique, and we believe a very valuable feature of the book is the report of the author's investigations of pistol-shot wounds of the cranium. This is a record of the external appearance of wounds made by various sized pistol-balls fired at different distances from the body. To make this part of the book more interesting and attractive, a large number of illustrations made from photographs of persons who had recently received pistol-shot wounds, are inserted. These are especially valuable in a medico-legal way since they demonstrate the character of the wound, the amount of powder burn or stain, etc., when the shot has been fired at different distances from the body.

The book, as a whole, is very instructive and contains much important matter which cannot be had elswhere. S. E. H.

